# Protocol for isolation of human bone marrow stromal cells and characterization of cellular metabolism

**DOI:** 10.1016/j.xpro.2024.103553

**Published:** 2025-01-13

**Authors:** Martina Dzubanova, Michaela Ferencakova, Andrea Benova, Dalia Ali, Michaela Tencerova

**Affiliations:** 1 Laboratory of Molecular Physiology of Bone, Institute of Physiology of the Czech Academy of Sciences, Prague, Czech Republic; 2 Faculty of Science, Charles University, Prague, Czech Republic; 3 Department of Endocrinology and Metabolism, Molecular Endocrinology & Stem Cell Research Unit (KMEB), Odense University Hospital, University of Southern Denmark, Odense, Denmark

**Keywords:** Cell Biology, Metabolism, Stem Cells

## Abstract

Bone marrow stromal cells (BMSCs) serve as a valuable reservoir of multipotent stem cells important in the regulation of bone homeostasis and energy metabolism. Here, we present a protocol for isolating human BMSCs (hBMSCs) and characterizing their cellular metabolism related to hBMSC functional properties. We describe steps for bioenergetics, cell senescence, and production of reactive oxygen species (ROS), together with description of the data analysis. These assays provide information on hBMSC metabolic status valuable to regenerative medicine and therapeutic applications.

For complete details on the use and execution of this protocol, please refer to Tencerova et al.^1^

## Highlights

• Isolation and cultivation of hBMSCs from bone marrow aspirates
• Steps to measure hBMSC bioenergetics, ROS production, and senescent phenotype
• Data analysis of metabolic assays and troubleshooting

## Abstract (editor highlights)

Publisher’s note: Undertaking any experimental protocol requires adherence to local institutional guidelines for laboratory safety and ethics.

## Graphical abstract

**Figure:**
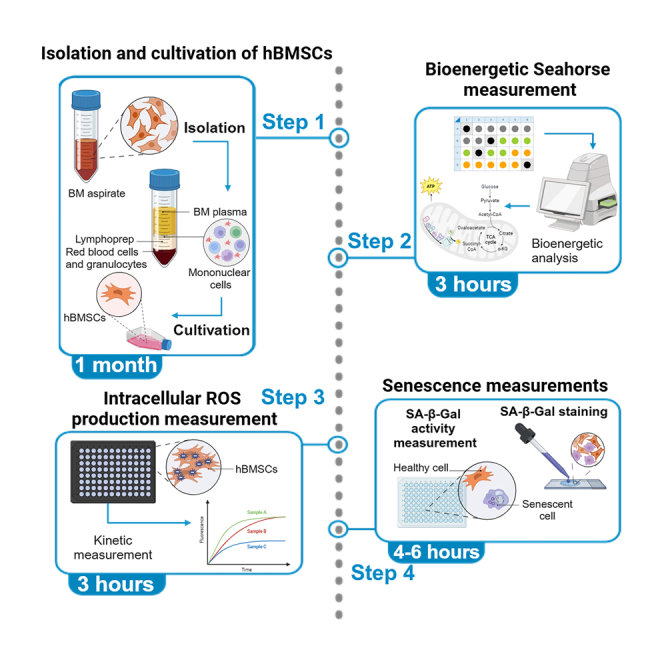

## Before you begin

Bone marrow stromal cells (BMSCs) are a source of multipotent stem cells, which play an important role in the regulation of bone homeostasis and energy metabolism and represent a key source for tissue engineering, regenerative medicine, and diagnosis of metabolic and bone diseases. Previous studies, including our findings,^1^^,^^2^^,^^3^^,^^4^^,^^5^^,^^6^ demonstrated that the measurement of cellular metabolism including bioenergetics, production of reactive oxygen species (ROS), and senescence, among others is a critical feature to evaluate the stem cell potency and functional capacity of BMSCs in bone metabolism and regenerative processes in connection to different physiological and pathophysiological conditions.

In the main manuscript connected to this STAR protocol,^1^ we reported that obesity causes hyperactivation of the metabolic status of human BMSCs (hBMSCs), characterized by upregulation of insulin signaling associated with enhanced mitochondrial respiration and ROS production, contributing to a senescent phenotype of hBMSCs. Thus, targeting cellular metabolism in metabolic and bone diseases has increased interest among researchers to develop new therapeutic approaches to regulate BMSC metabolism. Even though cellular metabolism can reflect the changes in cell proliferation, differentiation potential, cell survival, or inflammatory status, several advanced techniques can be applied to measure metabolic status of the cells. However, working with clinical material can limit how many different methods you can use to evaluate stem cell metabolism.

Here, we present a step-by-step protocol for hBMSC isolation and characterization of hBMSC cellular metabolism, which we optimized for the measurement of bioenergetic profile, ROS production, and senescence, including tips for successfully setting up the protocol and troubleshooting of specific methods.

Before initiating the isolation of hBMSCs from human bone marrow (BM) aspirates, several critical steps need to be considered to ensure the ethical, logistical, and methodological aspects are well addressed. These preparatory steps contribute to the reliability and reproducibility of the experimental procedures while upholding ethical standards.

### Institutional permissions

This protocol requires human samples obtained from patients undergoing a BM aspirate procedure. All BM aspirates were collected according to the Declaration of Helsinki. Ethical approval was obtained from the ethical committee and informed consent was obtained from all subjects (IORG0002175, IRB00002705, FWA00029052) of The General University Hospital in Prague (156/22 S-IV).

Before you start working with human BM samples, you need to prepare:

1. Ethical approval and informed consent:
  a. Ensure that all experiments involving human tissue have received approval from the national ethics committee.
  b. Confirm that informed consent has been obtained from all patients participating in the study, in accordance with ethical guidelines.
2. Collaboration with surgeons:
  a. Establish a collaborative relationship with surgeons to facilitate the collection of BM samples.
  b. Coordinate with the surgical team to organize sample collection from patients undergoing BM biopsy.
  c. Ensure that BM aspirates are collected in a consistent manner to avoid any discrepancies in the procedure.^7^
3. Prepare samples for transport:
  a. Prepare a transport bag the day before BM aspiration.
  b. Add heparin (an anticoagulant) to a 50 mL falcon tube with MEM medium and store in the fridge (see materials and equipment).
  c. Transport BM samples at 20°C–23°C.

***Note:*** The method for hBMSC isolation involves BM collecting into tubes containing an anticoagulant, which can potentially affect cell characteristics. To determine the suitable anticoagulant, refer to our study on how anticoagulants influence hBMSCs molecular properties.^8^

4. Sterilize all equipment in advance (see materials and equipment).
5. Ensure an adequate amount of stock solutions is available for all planned procedures.
6. Note that some solutions must be freshly prepared before cell isolation (see materials and equipment).

## Key resources table

**Table:** 

REAGENT or RESOURCE	SOURCE	IDENTIFIER
**Biological samples**		

Bone marrow aspirate	Orthopedic surgery unit	N/A

**Chemicals, peptides, and recombinant proteins**		

β-mercaptoethanol	Merck	63689
2-deoxy-D-glucose >= 98%	Merck	D8375-100G
Antimycin A from *Streptomyces* sp.	Merck	A8674-25MG
D-(+)-Glucose >= 98%	Merck	68270-100G
Dimethylformamide (DMF)	Cell Signaling Technology	12767
Dimethyl sulfoxide (DMSO) Hybri-Max sterile-filtered	Merck	D2650-5X10ML
Dulbecco’s modified Eagle’s medium	Merck	D5030-10x1L
Dulbecco’s PBS (1×), w/o Ca & Mg, w/o		
Dulbecco’s PBS with MgCl_2_ and CaCl_2_	Merck	D8662
FCCP = carbonyl cyanide 4-(trifluoromethoxy)phenylhydrazone	Merck	C2920
Fetal bovine serum	Capricorn	FBS-11A F
GlutaMAX supplement	Invitrogen	35050038
Glycerol	Sigma-Aldrich	G5516
Heparin (5,000 U/mL)	Pharmacy (Zentiva)	12149
Hoechst 33342	Thermo Fisher Scientific	H1399
Lymphoprep	STEMCELL Technologies	07851
MEM media	Invitrogen	31095
MEM non-essential amino acids solution	Invitrogen	11140035
Oligomycin from *Streptomyces diastatochromogenes*	Merck	O4876-5MG
Penicillin-streptomycin solution	Invitrogen	15140130
Phosphate-buffered saline	Invitrogen	14190169
Proteinase inhibitor cocktail (PIC)	Roche	11873580001
Rotenone	Merck	R8875-1G
Sodium pyruvate	Invitrogen	11360039
Trypan blue 0.4%	Merck	T8154-100ML
Trypsin 0.05% EDTA	Invitrogen	25300062

**Critical commercial assays**		

96-well cellular senescence assay kit, SA-β-gal activity	Cell Biolabs, Inc.	CBA-231
BCA protein assay kit	Merck	71285-3
DCFDA/H2DCFDA - cellular ROS assay kit	Abcam	ab113851
Senescence β-galactosidase staining	Cell Signaling Technology	9860

**Software and algorithms**		

Wave 2.6.1.53	Agilent Technologies	https://www.agilent.com/cs/library/software/public/ReadMe_Wave_Desktop_2-6.pdf
GraphPad Prism 9.5.1	Dotmatics	https://www.graphpad.com/features
Microsoft Excel	Microsoft	https://www.microsoft.com/en-us/microsoft-365/excel

**Other**		

20 mL syringes	CHIRANA T.Injecta	J54-075
96-well clear plates with flat bottom for cell culture	TPP	92696
96-well black plate with clear flat bottom for cell culture	Brand (VWR)	735-2104 781971
BioTek Cytation 3 cell imaging multi-mode reader	BioTek	N/A
Centrifuge 3-16KL	Sigma	10360
Falcon tubes (15 mL and 50 mL)	Sarstedt	62.554.502 62.547.254
IKA Vortex 3	Schoeller	N/A
Microcentrifuge tubes for 1.5–2 mL	Sarstedt	72.706
Microscope	Leica Microsystems	N/A
Needles 1.2 × 40 mm 18G × 1 1/2″	Henke Sass Wolf	4710012040
Needles 2.00 × 120 mm 14 G × 4 3/4″	CHIRANA T.Injecta	CH14434
Non-sterile incubator 37°C without CO2	P-LAB	N/A
Nunc CryoTube vials 1.8 mL	Thermo Fisher Scientific	177280
Pasteur glass pipettes	Hirschmann	H925101
Pipette rubber teats	P-LAB	R353161
Seahorse XFe24 FluxPak	Agilent	102340-100
Seahorse XFe24 analyzer	Agilent Technologies	N/A
Sterile incubator 37°C with CO_2_	Schoeller	N/A
Water bath JULABO SW22	JULABO	N/A

***Alternatives:*** The chemicals, instruments and software mentioned above are from specific brands that we prefer in our laboratory. We encourage readers to explore other brands available on the market to determine, which options are most sensitive and robust for their experiments.

## Materials and equipment

**Table:** Heparinized MEM media

Reagent	Final concentration	Amount
MEM media	N/A	9 mL
Heparin (5000 U/mL)	500 U/mL	1 mL

Prepare a sterile solution and keep in 50 mL Falcon tube. Store at 4°C.

***Note:*** 1 tube with 10 mL corresponds to BM aspirate from 1 donor.

**Table:** hBMSC isolation media

Reagent	Final concentration	Amount
MEM media	N/A	89 mL
FBS	10%	10 mL
Penicillin-Streptomycin Solution (Pen/Strep) (10,000 Units/mL Penicillin; 10,000 μg/mL Streptomycin)	1% (100 Units/mL Penicillin; 100 μg/mL Streptomycin)	1 mL

Prepare a sterile solution and store at 4°C.

**Table:** hBMSC cultivation media

Reagent	Final concentration	Amount
MEM media	N/A	86 mL
FBS	10%	10 mL
Pen/Strep (10 000 Units/mL Penicillin; 10 000 μg/mL Streptomycin)	1% (100 Units/mL Penicillin; 100 μg/mL Streptomycin)	1 mL
GlutaMAX Supplement (200 mM)	2 mM	1 mL
Sodium Pyruvate (100 mM)	1 mM	1 mL
MEM Non-Essential Amino Acids Solution (100×)	1×	1 mL

Prepare a sterile solution and store at 4°C.

**Table:** hBMSC freezing media

Reagent	Final concentration	Amount
FBS	80%	800 μL
DMSO	10%	100 μL
MEM media	10%	100 μL

**CRITICAL:** Prepare fresh before use.

**Table:** BCA working reagent

Reagent	Final concentration	Amount
BCA solution	N/A	200 μL
4% Cupric Sulfate	N/A	4 μL

All reagents are included in the BCA protein assay kit.

**Table:** Basal media

Reagent	Final concentration	Amount
Dulbecco's Modified Eagle's Medium	8.3 g/L	dissolve 415 mg in 48 mL ultra-pure sterile dH_2_O
D-(+)-Glucose >= 98% (2 M)	10 mM	0.25 mL
GlutaMAX Supplement (200 mM)	4 mM	1 mL
Sodium Pyruvate (100 mM)	2 mM	1 mL

**CRITICAL:** Warm the media at least 45 min before incubation with the cells to 37°C and adjust its pH to a final value of 7.4 ± 0.05.

***Optional:*** Instead of DMEM powder, there is an option to use Agilent Seahorse XF DMEM medium, pH 7.4, (500 mL) without phenol red.

**Table:** D-(+)-Glucose >= 98%

Reagent	Final concentration	Amount
D-(+)-Glucose >= 98% (2 M)	100 mM	add 100 μL to 1.9 mL basal media

Prepare fresh before use.

**Table:** Oligomycin from Streptomyces diastatochromogenes (Oligomycin)

Reagent	Final concentration	Amount
Oligomycin (5 mM)	10 μM	add 4 μL to 2 mL basal media

Prepare fresh before use.

**Table:** Carbonyl cyanide 4-(trifluoromethoxy)phenylhydrazone (FCCP)

Reagent	Final concentration	Amount
FCCP (5 mM)	20 μM	add 8 μL to 2 mL basal media

Prepare fresh before use.

**Table:** 2-deoxy-D-Glucose>= 98% (2-DG)

Reagent	Final concentration	Amount
2-DG	1 M	dissolve 1.67 g in 10 mL basal media

Prepare 2 mL aliquots and store at −20°C.

**Table:** Rotenone/Antimycin A from Streptomyces sp. (Antimycin A)/Hoechst

Reagent	Final concentration	Amount
2-DG	1 M	2 mL
Rotenone (5 mM)	10 μM	4 μL
Antimycin A (5 mg/mL)	10 μg/mL	4 μL
Hoechst 33342 (1,000×)	10×	20 μL

Prepare fresh before use. Thaw 2-DG aliquot at 20°C–23°C.

***Optional:*** Skip the addition of Hoechst if you use different method for normalization of the Seahorse measurement (e.g., protein or DNA). If using Hoechst, protect the plate from light exposure during the experiment.

**Table:** Intracellular ROS production measurement components

Reagent	Final concentration	Amount
Buffer (10×)	1×	add 1 mL 10× Buffer to 9 mL ultra-pure sterile dH_2_O
Supplemented Buffer	N/A	add 1 mL FBS to 9 mL 1× Buffer
2′,7' –dichlorofluorescein diacetate (DCFDA) (20 mM)	25 μM	add 1.25 μL of DCDFA to 1 mL 1× Buffer
tert-Butyl hydroperoxide (TBHP) solution (55 mM)	50–100 μM	add 1.8 μL of TBHP to 998.2 μL 1× Supplemented Buffer

All reagents, except Supplemented Buffer, are included in the DCFDA / H2DCFDA - Cellular ROS Assay Kit. Store the kit at 4°C in the dark. For longer-term storage (over 3 months), store the kit at −20°C. For more information on the storage of individual components, please refer to the manufacturer’s manual (https://www.abcam.com/en-us/products/assay-kits/dcfda-h2dcfda-cellular-ros-assay-kit-ab113851#tab=support).

***Note:*** The exact concentration of DCFDA and TBHP required will depend on the cell type used; a general starting range is 10–100 μM.

**CRITICAL:** Equilibrate the 1× Buffer to 37°C before use. Prepare fresh Supplemented buffer, TBHP, and DCFDA solution before each use.

***Optional:*** 1× Buffer can be kept frozen at −20°C for future use. DCFDA and TBHP may be diluted in media without phenol red.

**Table:** Senescence measurement components

Reagent	Final concentration	Amount
Lysis Buffer	N/A	mix 0.33 mL 2× Lysis Buffer with 0.67 mL dH_2_O and 40 μL Protease inhibitor cocktail (PIC)
Assay buffer	N/A	add 1 μL 10 M β-mercaptoethanol and 50 μL 20× SA-ß-Gal Substrate to 950 μL 2× Reaction Buffer

All reagents, except β-mercaptoethanol and PIC, are included in the 96-well Cellular Senescence Assay Kit, SA-β-gal activity.

***Optional:*** The diluted solution of Lysis Buffer can be stored at 20°C–23°C for up to six months without protease inhibitors.

**CRITICAL:** Immediately before using the Lysis Buffer, add the appropriate amount of PIC. Do not store the Assay buffer after adding SA-β-Gal substrate.

***Note:*** The amount of β-mercaptoethanol added to Reaction Buffer will depend on the concentration of your stock solution.

**Table:** Components for senescence β-Galactosidase Staining

Reagent	Final concentration	Amount
Fixative Solution (10×)	1×	mix 100 μL 10× Fixative Solution with 900 μL dH_2_O
Staining Solution (10×)	1×	mix 100 μL 10× Staining Solution with 900 μL dH_2_O
X-Gal	20 mg/mL	dissolve 20 mg X-Gal in 1 mL DMF

All reagents, except DMF, are included in the Senescence β-Galactosidase Staining Kit. Warm the staining solution at 37°C with gentle agitation.

**CRITICAL:** Prepare all solutions fresh prior to use. Store X-Gal solution in polypropylene plastic or glass containers.

***Optional:*** Excess X-Gal solution can be stored in −20°C, protected from light.

**Table:** β-Galactosidase Staining Solution

Reagent	Amount
1× Staining Solution	930 μL
100× Solution A	10 μL
100× Solution B	10 μL
X-Gal (20 mg/ml)	50 μL

**CRITICAL:** Adjust the pH of the β -Galactosidase Staining Solution to 6 using an acidic solution.

## Step-by-step method details

### BM sample collection and transport

**Timing: 2 h**

This section describes the collection and transport of BM aspirate.

**Figure 1:**
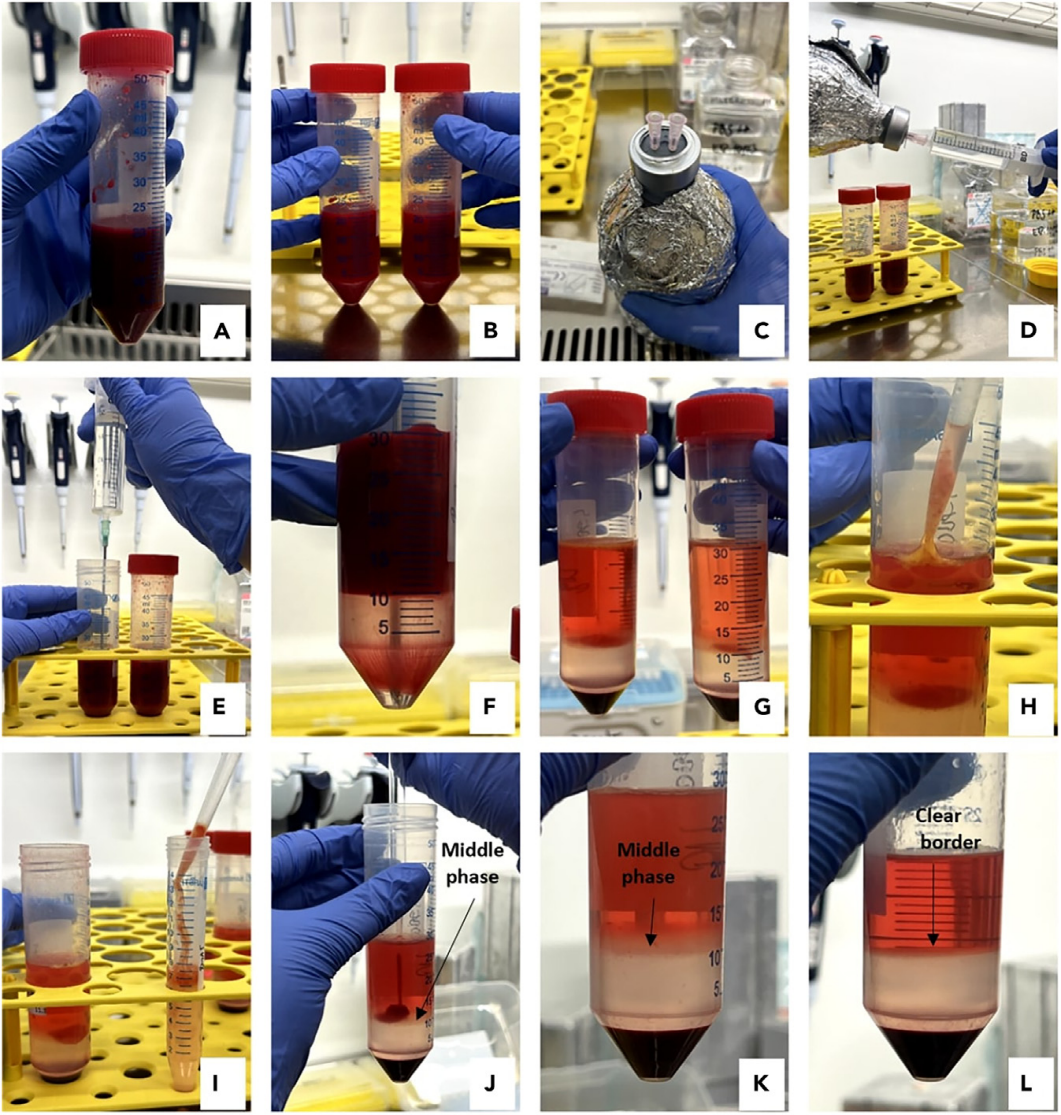
Isolation of hBMSCs from BM aspirates Obtained sample of BM aspirate (A); BM aspirate divided into two 50 mL Falcon tubes (B); Lymphoprep bottle with two small disposable needles (C); Lymphoprep aspiration using a small needle (D); Application of Lymphoprep using a kwill needle (E–F); Separated phases after centrifugation (G); Collection of BMAT into PBS (H–I); Middle phase aspiration (J–K); Clear borderline created after cell aspiration (L).

1. BM aspiration from the iliac crest performed by the surgeon
  a. Patients were administered a local anesthetic (lidocaine, 10 mg/mL) and a 5–10 mL BM sample was collected by the surgeon from the iliac crest into a 50 mL falcon tube containing heparinized MEM.
2. Bring the sample back to the laboratory within 2 h for processing (Figure 1A).

***Note:*** After collection, keep the samples at 20°C–23°C until transported to the lab.

### hBMSCs isolation

**Timing: 2 h**

This section describes the cell isolation of low-density mononuclear cells through centrifugation using a Lymphoprep density gradient (density = 1.077 ± 0.001 g/mL) and cell cultivation through the plastic adherence.

3. Dilute the BM aspirate 1:1 in PBS (with CaCl_2_ and MgCl_2_) and mix gently.
4. Divide the diluted aspirate equally between two 50 mL Falcon tubes (Figure 1B).
5. Using two small disposable needles, aspirate 10 mL of Lymphoprep.
6. Replace the needle with a kwill plastic needle and place the needle at the bottom of the BM-filled tube, and gently add 10 mL of Lymphoprep each falcon tubes (Figures 1C–1F).
7. Centrifuge the samples at 2 500 rpm (573 g) for 25 min at 20°C–23°C without brakes.
  a. After the centrifugation, a gradient forms, a layer of mononuclear cells on the top of Lymphoprep (Figure 1G).
8. Prepare a 50 mL Falcon tube with 20 mL of preheated hBMSC isolation media.
9. Using a glass Pasteur pipette and rubber teats, aspirate the middle phase (the narrow, turbid layer between Lymphoprep and the mononuclear cells) and transfer it to the prepared 50 mL Falcon tube containing 20 mL of hBMSC isolation media.
  a. Collect the middle phases from both tubes (Figures 1J and 1K). At the end, you should see a clear boundary between the phases (Figure 1L).
    **CRITICAL:** If a red clot is present in the middle phase, harvest it along with the narrow turbid phase (Figure 1J).
    ***Optional:*** At this step, you may collect the floating layer of mature bone marrow adipose tissue (BMAT) on top (Figures 1H and 1I). If harvesting BMAT, do so before hBMSC collection to avoid disrupting the phase.
  b. If BMAT does not pass through the pipette tip, cut the top of the tip with sterile scissors to increase its diameter.
  c. Wash the BMAT with PBS.
  d. Prepare aliquots for RNA (mix BMAT with Trizol) or protein (mix BMAT with lysis buffer), and store at −80°C for subsequent analysis.
10. Centrifuge the tube containing the middle phase at 2 000 rpm (367 g) for 10 min at 20°C–23°C.
11. Aspirate the clear supernatant and resuspend the cells in fresh hBMSC isolation media (∼10–20 mL).
12. Count the cells using 0.4% trypan blue (dilution 1:1).
13. Seed the cells at a density of 10 mil. per T-75 flask in 10 mL of hBMSC isolation media and cultivate the cells in a sterile incubator (37°C and 5% CO_2_).

**CRITICAL:** Do not change the media for one week (7 days) to allow the hBMSCs adhere to the plastic.

### hBMSC culture and seeding for analysis

**Timing: 1 month**

This section describes the process of maintaining and expanding hBMSCs, ensuring their viability and consistency for downstream applications. It includes instructions for media changes, cell passaging, and freezing, which are critical for preserving cell integrity and reproducibility and instructions for seeding cells for Seahorse, ROS production and senescence measurements.

14. After 7 days from cell isolation, aspirate isolation media and add a fresh hBMSC cultivation media and change the media every 2–3 days.
15. When cells reach 80% confluence, expand hBMSCs to the next passage or seed for subsequent analysis.
  **CRITICAL:** Maintaining proper cell growth is essential. The time required for isolated hBMSCs to reach confluence depends on the individual BM samples. However, hBMSC should not remain in passage 0 (p0) for more than 1 month. After this period, the cells should either be passaged to p1 or frozen in liquid nitrogen (LN_2_).
  **Pause point:** Once hBMSCs reach confluence in p0, they can either be expanded or frozen in LN_2_ for later metabolic assays. At this stage, you can pause the experiment and continue with further hBMSC measurements but make sure to keep it consistent for all samples.
  a. Passaging hBMSCs:
    i. Aspirate the cell culture media from the T-75 flask.
    ii. Wash the cells with 5–7 mL of sterile PBS and incubate for 5 min at 37°C with 5% CO_2_.
    iii. Aspirate the PBS and add pre-warmed Trypsin/EDTA to detach the cells (Note).
    iv. Incubate the cells with Trypsin/EDTA for 5 min at 37°C with 5% CO_2_.
    v. Once the cells have detached, add the pre-warmed complete hBMSC cultivation media (the same volume as Trypsin to stop the enzymatic reaction).
    vi. Mix the cell suspension in the flask and transfer to a 50 mL falcon tube and centrifuge at 1 200 rpm (132 g) for 5 min at 20°C–23°C.
    vii. Resuspend the cell pellet in an appropriate volume of pre-warmed hBMSC cultivation media, depending on the pellet size, and count the cells.
      ***Note:*** The volume of Trypsin depends on the size of the flask (3 mL in T75, or 5 mL in T150).
  b. Freezing hBMSCs:
    i. After counting cells prepare an appropriate amount of hBMSC freezing media (1 CryoTube: 1 mil. cells in 1 mL of hBMSC freezing media).
    ii. Centrifuge the cell suspension at 1 200 rpm (132 g) for 5 min at 20°C–23°C.
    iii. Resuspend the pellet in hBMSC freezing media and transfer to CryoTubes.
    iv. Store at −80°C for 24 h, then transfer CryoTubes to LN_2_ for long-term storage.
      ***Note:*** Avoid repeated freeze-thaw cycles by using each aliquot only once.
  c. Cell seeding for seahorse measurement:
    i. Mark the background wells on the XF24 cell culture microplate cover with an “X” symbol (wells 1A, 3B, 2C, and 6D) (Figure 3C).
    ii. Seed cells in the remaining wells of the XF24 cell culture microplate at a density of 20 000 cells/well (ideally 3-5 replicates per condition) in 100 μL/well of hBMSC culture media.
    iii. After approximately 3 h, add an additional 150 μL/well of the same media.
  d. Cell seeding for intracellular ROS production measurement:
    i. Seed cells into a sterile black 96-well plate with a clear flat bottom at a density of 15 000 cells/well in 200 μL/well of cell culture media (ideally 5-6 replicates per condition).
    ii. Include at least two positive control wells, reserved for TBHP treatment, containing cells but no test compounds.
  e. Cell seeding for senescence measurement:
    i. Seed cells at a density of 10 000 cells/well in 200 μL/well in a sterile, clear 96-well plate with a flat bottom. Seed 5-6 replicates per condition.
  f. Cell seeding for senescence β-Galactosidase staining:
    i. Seed cells at a density of 15 000 cells/well in 200 μL/well in a sterile, clear 96-well plate with a flat bottom.
      **CRITICAL:** The passage number of the cells must be consistent across different experimental trials to ensure reproducibility.
      ***Optional:*** If the cells are prone to detachment, consider coating the plate with (e.g. with poly-K, Cell-Tak etc.)
      ***Optional:*** Before any of the measurements mentioned above, you may apply treatments to the cells either the day before or immediately prior to the measurement, depending on whether you aim to observe long-term or acute effects of drugs, inhibitors, etc.

### Seahorse measurement

**Timing: 3 h**

The Seahorse measurement evaluates the oxygen consumption rate (OCR) and extracellular acidification rate (ECAR) at intervals of minutes. OCR, reported in pmol/min, reflects mitochondrial respiration, while ECAR, reported in mpH/min, indicates glycolysis. This protocol provides a way to measure these metabolic processes simultaneously in one measurement. The main components for Seahorse measurement are included in the Seahorse XFe24 FluxPak (See materials and equipment), which provides 18 XFe24 sensor cartridges, 20 XF24 cell culture microplates, and 1 bottle of Seahorse XF Calibrant Solution (500 mL). Additional chemicals are listed in the materials and equipment section.

16. On the day of cell seeding, hydrate the XFe24 sensor cartridge.
  a. Remove the lower Utility Plate from the cartridge and add 1 mL/well of Agilent Seahorse XF Calibrant.
  b. Place the cartridge in the incubator without CO_2_ set to 37°C.

**CRITICAL:** Ensure the incubator has a water source (either a tank or a beaker of distilled water) to maintain humidity during cartridge hydration.

**Pause point:** If the cells are not ready, you can delay the measurement. Cartridge hydration should be valid between 4 h and 72 h. Optimal is approx. 15 h (overnight).

**Figure 2:**
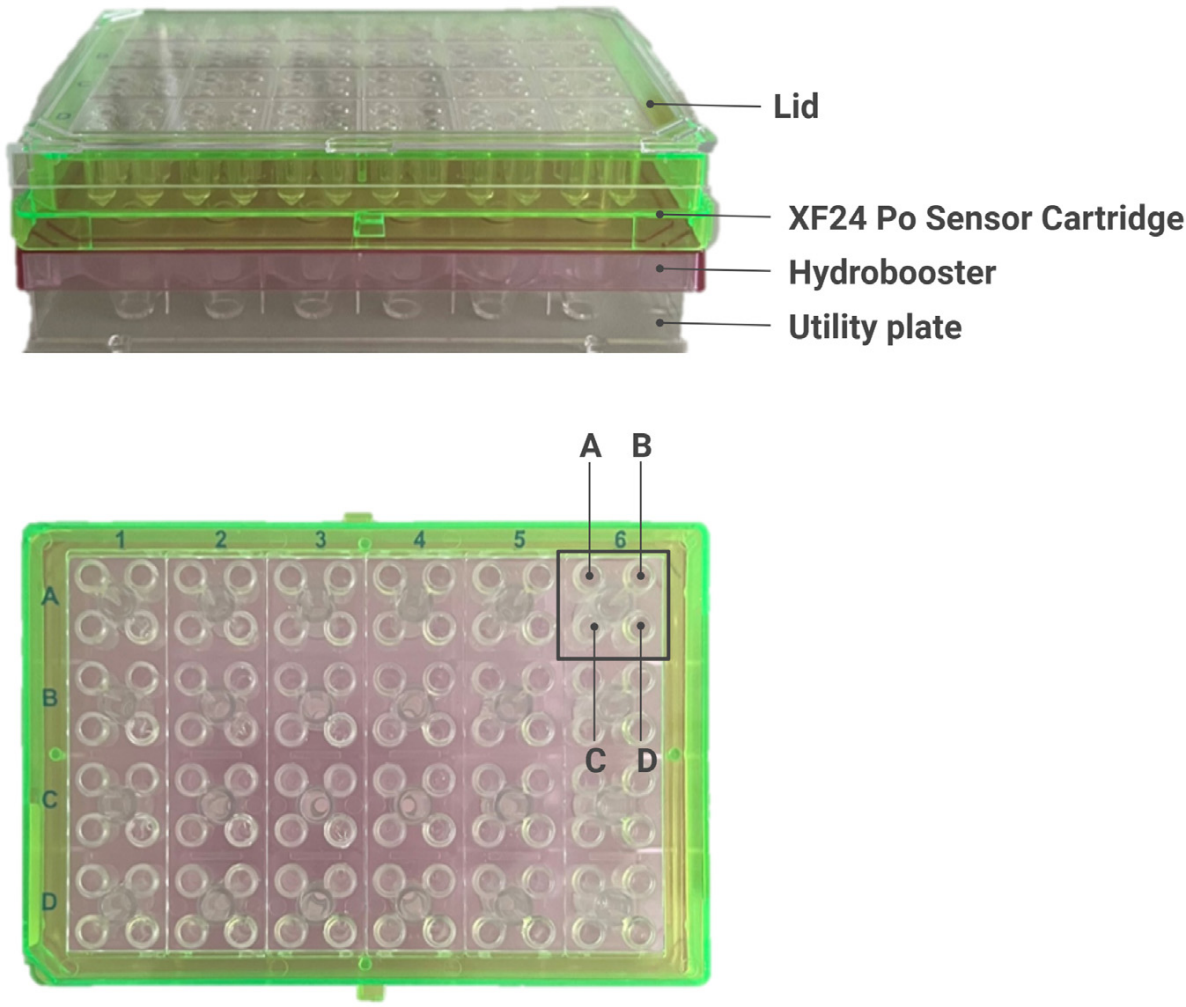
Assembly of the Agilent Seahorse XF24 The Agilent Seahorse XF24 consists of a Hydrobooster, an Agilent Seahorse XF24 Pro sensor cartridge with four ports (A–D), and utility plate. Created in BioRender (https://BioRender.com/h56e751).

17. Turn on the Seahorse analyzer and launch the Wave software at least 4 h before the measurement to preheat the instrument.
18. Prepare basal media and assay components as described in materials and equipment.
19. Rinse each well of the XF24 cell culture microplate containing cells with 1 mL/well of basal media.
20. Aspirate and add 500 μL/well of basal media (including backgrounds wells).
21. Incubate cells in the incubator without CO_2_ for 30–60 min.
22. Add assay components to the cartridge ports (including backgrounds wells) (Figure 2):

**Figure 3:**
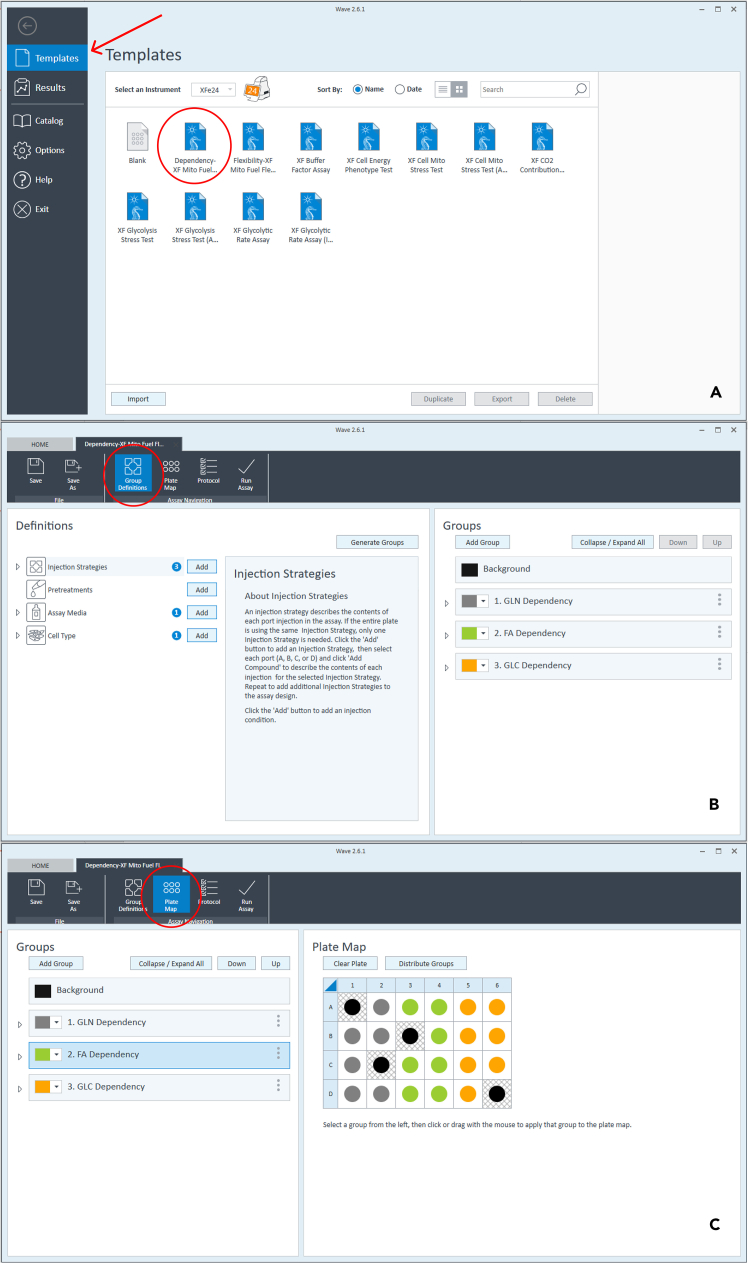
Measurement settings in Wave software Wave software preview (A); Group Definitions (B). Plate map and background settings (C).

**Table 1:** Example of template used for simultaneous measurement of OCR and ECAR, related to step 23

Port	Duration	Cycles	Mix	Wait	Measure
A - Glucose	00:21:00	3	03:00	01:00	03:00
B - Oligo	00:21:00	3	03:00	01:00	03:00
C - FCCP	00:24:00	3	03:00	02:00	03:00
D - 2-DG/Rot/AA	00:18:00	3	02:00	01:00	03:00

Port A – 55 μL.

Port B – 60 μL.

Port C – 68 μL.

Port D – 76 μL

***Optional:*** The order of compounds may vary depending on your Seahorse protocol. This setup combines components for glycolysis and respiration, allowing simultaneous measurement. For alternative protocols or separate metabolic pathways analysis, Agilent XF Assay Kits and Reagents offer other options with specific instructions.

**CRITICAL:** Do not centrifuge the cartridge with assay components, as reagents may leak from the ports.

**CRITICAL:** Prepare assay components fresh before measurement. Components from ports A-D are added during the measurement. Therefore, in our case, the final concentration used during measurement in the wells are:

Port A – 10 mM Glc.

Port B – 1 μM OLIGO.

Port C – 2 μM FCCP.

Port D – 1 μM Rotenone + 1 μg/mL Antimycin A in 2-DG + 1X Hoechst.

23. Measurement setup in Wave software:
  a. In the Wave software (Figure 3A), open “Templates” for measurement and choose “Group Definitions” to setup the measurement (Figure 3B).
  b. Open the plate map, designate background wells, and select replicate wells based on your experimental design.
  c. Click “Run Assay” to initiate calibration (Figure 3C).

***Note:*** An example template is provided in Table 1.

24. Insert the prepared cartridge with assay components and start calibration.

**CRITICAL:** Remove the pink hydrobooster and lid (Figure 2).

25. Once calibration is complete, replace the utility plate with the cell plate and start measurement.
26. After the measurement (duration depends on your Protocol setup), proceed with normalization.

***Note:*** Normalization of the Seahorse data is especially important for primary cells, where cell populations may be heterogeneous. There are several options how to normalize Seahorse measurement based on laboratory preferences.

27. Seahorse normalization methods
  a. Cell count
    i. For cell counting, use the BioTek Cytation 3 Cell Imaging Multi-Mode Reader.
      ***Optional:*** You can use other suitable imaging reader.
    ii. Perform cell counting, adjusting the threshold value as needed.
    iii. Export cell count data to Excel.
  b. BCA assay (Protein concentration analysis)
    i. After measurement, aspirate the media, rinse with PBS, and add 50 μL of lysis buffer for protein isolation.
    ii. Centrifuge the samples at 10 000 rpm (9 168 g) for 10 min at 4°C.
    iii. Pipette 10 μL of cell lysate into each well of a clear 96-well plate.
    iv. Add 200 μL of BCA working reagent to each well.
    v. Incubate the plate at 37°C for 30 min, protected from light.
    vi. Measure absorbance at 562 nm using a plate reader.

### Senescence and oxidative stress

These protocols outline the measurement of basal oxidative stress and senescence in hBMSCs, which can be used to assess induced oxidative stress and senescence^1^ and to test the impact of compounds and drugs on hBMSCs properties.^9^^,^^10^ When working with a different cell line, consider testing for optimal cell density, dose-response and viability.

### Intracellular ROS production measurement

**Timing: 3 h (may vary based on experiment setups)**

The DCFDA/H2DCFDA Cellular ROS Assay Kit measures ROS in adherent cells using the cell-permeant dye 2′,7′-dichlorofluorescin diacetate (DCFDA). Inside the cell, DCFDA is deacetylated by esterases to a non-fluorescent compound, which is then oxidized by ROS into the highly fluorescent 2′,7′-dichlorofluorescein (DCF), detectable by fluorescence spectroscopy. All components for ROS measurement are included in the DCFDA / H2DCFDA - Cellular ROS Assay Kit (See materials and equipment), which provides 20 mM DCFDA (in DMSO) 35 μL, 10× Buffer 10 mL and 55 mM TBHP 50 μL.

28. Remove the media and wash the cells with 100 μL/well of 1× Buffer or PBS.

***Note:*** Include blank wells without cells but with the test compound at the same concentration used for treatment. The mean value of the blank will be subtracted from the normalized well values after measurement.

29. Remove the 1× Buffer and stain cells by adding 100 μL/well of DCFDA Solution.
30. Incubate cells with DCFDA Solution for 45 min in a sterile incubator at 37°C with 5% CO_2_, protected from the light.
31. Remove the DCFDA Solution and wash the cells with 1× Buffer or PBS.
32. Add 100 μL/well of 1× Buffer or PBS.

***Optional:*** To test acute effect of specific compounds, replace the 1× Buffer or PBS with Supplemental buffer containing the test compound of interest or TBHP as a positive control (incubation duration depends on the cell type). After incubation, do not wash; measure fluorescence directly.

***Note:*** For our positive control setup, TBHP (100 μM) is incubated for 2 h in a sterile incubator at 37°C with 5% CO_2_ in the dark.

33. Measure fluorescence kinetics using a plate reader at Ex/Em = 485/535 nm every min for 30 min.

***Note:*** The optimal reading typically occurs between 10-15 min, as saturation may occur afterward. This may vary based on cell type or treatment.

***Alternatives:*** Mitochondrial ROS (mROS) is an important parameter to consider, especially when studying mitochondrial-specific oxidative stress and its implications in metabolic pathways. We encourage readers to consider to measure mROS assays available on the market, such as MitoSOX Red, a fluorogenic dye that selectively detects superoxide in mitochondria.^11^^,^^12^

### Senescence measurement

**Timing: 4–6 h**

Senescent cells exhibit markers like acidic senescence-associated β-galactosidase (SA-β-Gal) activity, which can be observed both in cultured cells and *in vivo*. The 96-well Cellular Senescence Assay Kit quantifies cellular senescence by measuring SA-β-Gal activity via a fluorometric substrate.

All components for Senescence measurement are included in the 96-well Cellular Senescence Assay Kit (See materials and equipment), which provides 2× Cell Lysis Buffer 10 mL, 2× Reaction Buffer 10 mL, 20× SA-β-Gal Substrate 300 μL, Stop Solution 25 mL.

34. Remove media from each well and wash cells with 100 μL of cold PBS.
35. Add 100 μL/well of cold prepared Lysis Buffer and incubate on ice for 5 min.
36. Scrape the cell lysate up and down in each well to ensure efficient cell lysis.
37. Transfer the cell lysate from each well into labeled microcentrifuge tubes and centrifuge at 10 000 rpm for 10 min at 4°C.
38. Measure protein concentration using the BCA assay (see Normalization of Seahorse measurement).
39. Carefully transfer 50 μL of cell lysate into a 96-well clear plate, add 50 μL/well of the Assay buffer, and incubate at 37°C (without CO_2_) for 3 h, protected from light.

***Optional:*** Incubation time may vary depending on cell type (typically 1–3 h).

40. After incubation, transfer 50 μL of the reaction mixture to a black 96-well plate with a clear flat bottom suitable for fluorescence measurement.
41. Measure fluorescence using a fluorescence plate reader at Ex/Em = 360/465 nm.

### Senescence β-Galactosidase staining

**Timing: 1–2 h initial setup; imaging from 6 to 12 hours post-incubation**

This protocol describes the steps for detecting and visualizing senescent cells using SA-β-gal staining. The method allows for the identification of senescent cells based on their enzymatic activity.

42. Remove the media and wash the cells with 100 μL/well of PBS.
43. Add 100 μL/well of 1× Fixative solution and incubate for 15 min.
44. Discard the 1× Fixative solution and wash the cells twice with 100 μL/well of PBS.

**Pause point:** Plates can be stored in PBS at 4°C.

45. Add 100 μL/well of β-Galactosidase Staining Solution.
46. Seal the plate with parafilm to prevent evaporation and incubate in a humidified incubator (without CO_2_) at 37°C.

**Figure 4:**
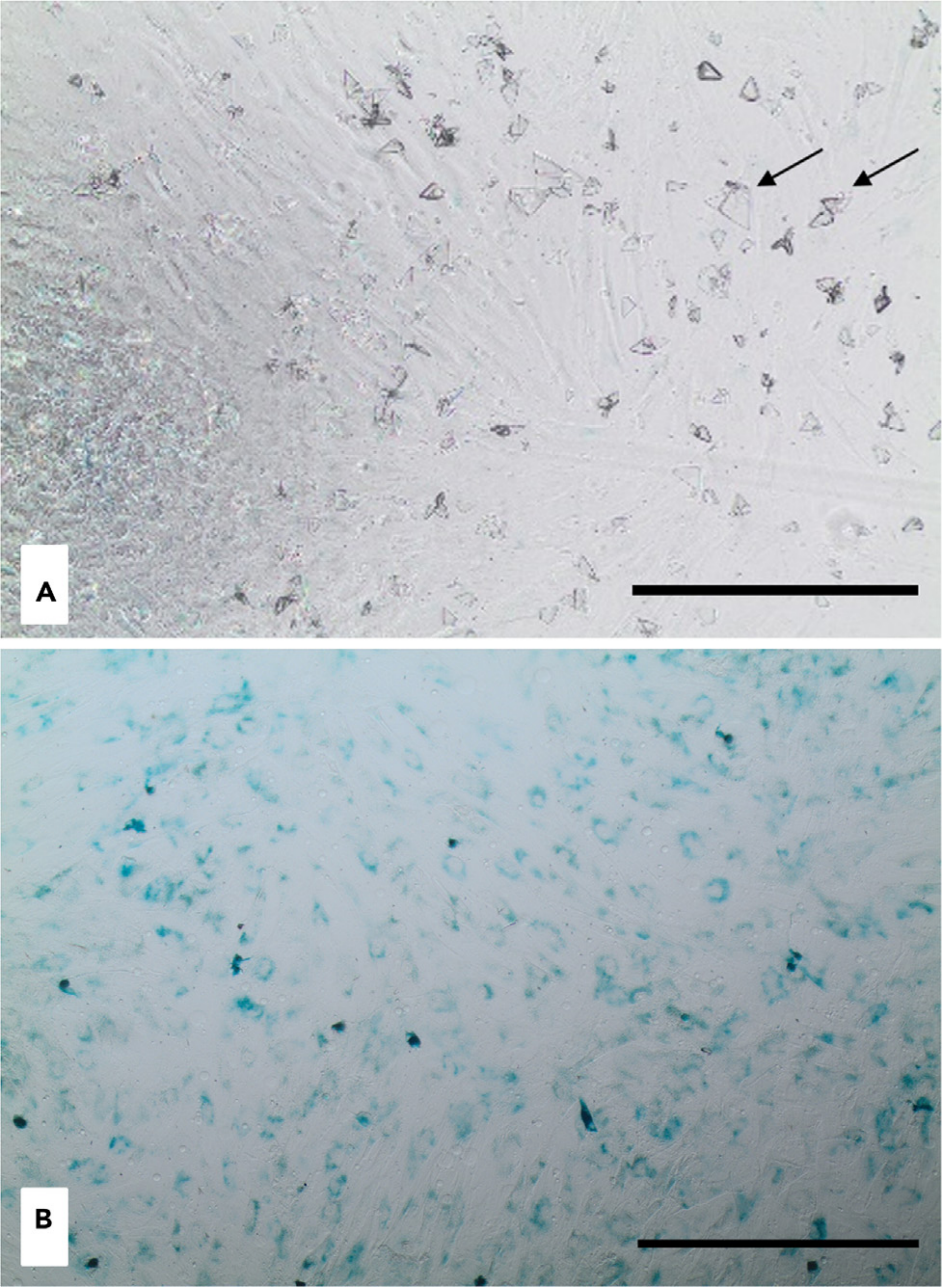
Senescence staining in hBMSCs Crystal formation during senescence staining: evaporation of the staining solution causes crystals formation (black arrows) (A); Senescence overstaining (B). Scale bar: 200 μm, magnification: 10×.

***Note:*** Avoid evaporation, as it may lead to crystals formation (Figure 4A). Also, CO_2_ can alter the pH, which is crucial for accurate characterization of the senescent cells.

47. Begin imaging with an inverted microscope at 20× magnification from 6 to 12 h post-incubation. Check for development of blue color in senescent cells.
  **CRITICAL:** Start imaging every hour between 6-12 h post-incubation to monitor senescent cell in comparison with positive and negative controls. Extended incubation may lead to all cells turning blue, which can result in false-positive staining if the β-Galactosidase Staining Solution oversaturates the cells (Figure 4B).
  ***Optional:*** For long-term storage, remove the β-Galactosidase Staining Solution and add 70% glycerol. Store plates at 4°C.
  ***Note:*** Quantify β-Galactosidase Staining by counting SA-β-gal-positive (blue) cells relative to total cells to calculate the percentage of SA-β-gal-positive cells, as described in.^13^^,^^14^
  ***Alternatives:*** Various methods are available for confirming cellular senescence, which may serve as additional verification of the senescent phenotype.^15^^,^^16^
  a. Detection of cell cycle genes pl6^Ink4a^, p21^Cipl^ via qPCR, western blot, or flow cytometry.
  b. Detection of Senescence-associated secretory phenotype (SASP), including pro-inflammatory cytokines, chemokines, growth modulators, angiogenic factors, and matrix metalloproteinases markers, via qPCR.
  c. Immunofluorescence measurement of γH2AX reflecting DNA damage.
  d. Measurement of Lamin B1 loss, a key component of the nuclear lamina that is downregulated in senescent cells.

## Expected outcomes

Using the methods described in this protocol, you can obtain between 2–15 mil. primary hBMSCs after initial *in vitro* expansion at p0. The final cell count will vary depending on factors such as the volume of the BM aspirate, donor age, sex, and health status. Expanded hBMSCs from p0 can be frozen in liquid nitrogen for future analysis or passaged for use in metabolic assays.

Seahorse measurement evaluates the bioenergetic profile of hBMSCs, including key parameters such as OCR and ECAR. This data provides an information about the current metabolic status of hBMSCs, including mitochondrial function, glycolytic activity, and total energy production capacity. These bioenergetics profiles help to understand how hBMSCs from different patients respond to physiological and pathological conditions, such as obesity, aging, or metabolic disorders.

ROS production measurement obtains quantitative data on intracellular ROS levels within hBMSCs, helping to determine oxidative stress – a key factor in cellular aging, senescence, and various metabolic dysfunctions. Elevated ROS levels can indicate mitochondrial dysfunction or other metabolic disturbances that may impair the regenerative potential of hBMSCs.

Senescence assays enable the detection and quantification of senescent cells within the hBMSC population by measurement of the markers such as SA-β-Gal activity. This data is crucial for understanding cellular aging within hBMSC cultures and can be correlated with other metabolic parameters, such as bioenergetic function and ROS production. High levels of senescence may suggest reduced stem cell potency and diminished capacity to support bone homeostasis and repair.

Together, these methods offer a comprehensive characterization of hBMSC metabolic function, oxidative stress levels, and senescence. These parameters are essential for evaluating the quality and functional potential of hBMSCs in both research and therapeutic contexts, particularly in the clinical studies of metabolic bone diseases.

## Quantification and statistical analysis

### Seahorse measurement

**Figure 5:**
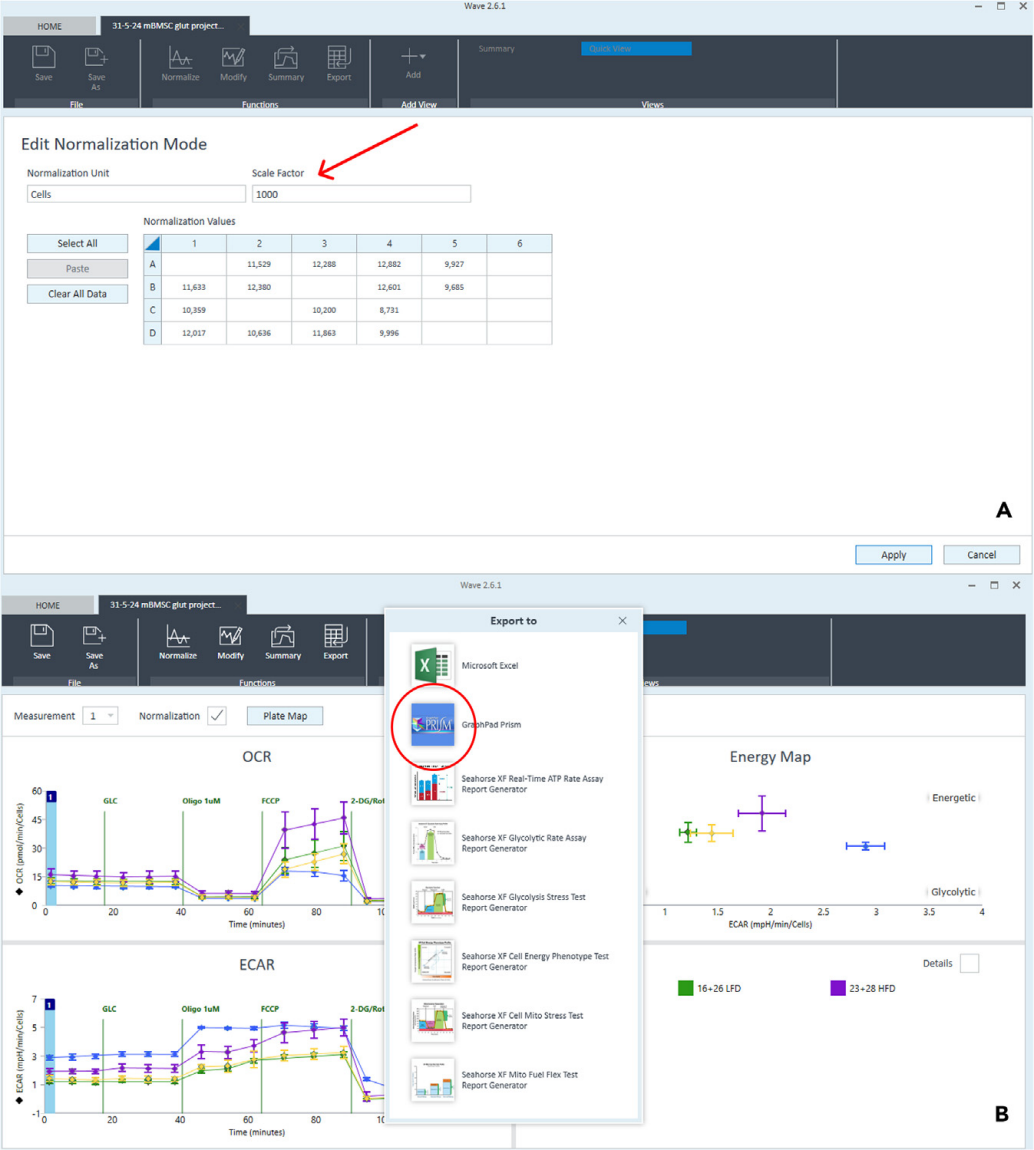
Data normalization and export in Wave software Normalization mode in Wave software (A) and Data export (B).

The analysis of Seahorse data involves multiple steps to process and interpret metabolic measurements. Get started with the raw data import into Wave software, followed by normalization using cell count data or protein concentration (Figure 5A). Apply the correct scale factor during this step (depends on your chosen normalization steps; e.g., a factor of 1 000 is often used for cell count normalization). Once normalized, check pH stability during the measurement by switching the "Display settings" above the graph in the software from "OCR" to "ECAR, and adjusting the grouping from "Group" to "Well" and "Rate" to "Level" to display pH values, which should remain near 7.4.

After confirming stable pH, export the results for further analysis (Figure 5B). Review individual well values for outliers by adjusting the display settings to show specific wells values. For more accurate analysis, only normalized data should be used.

**Figure 6:**
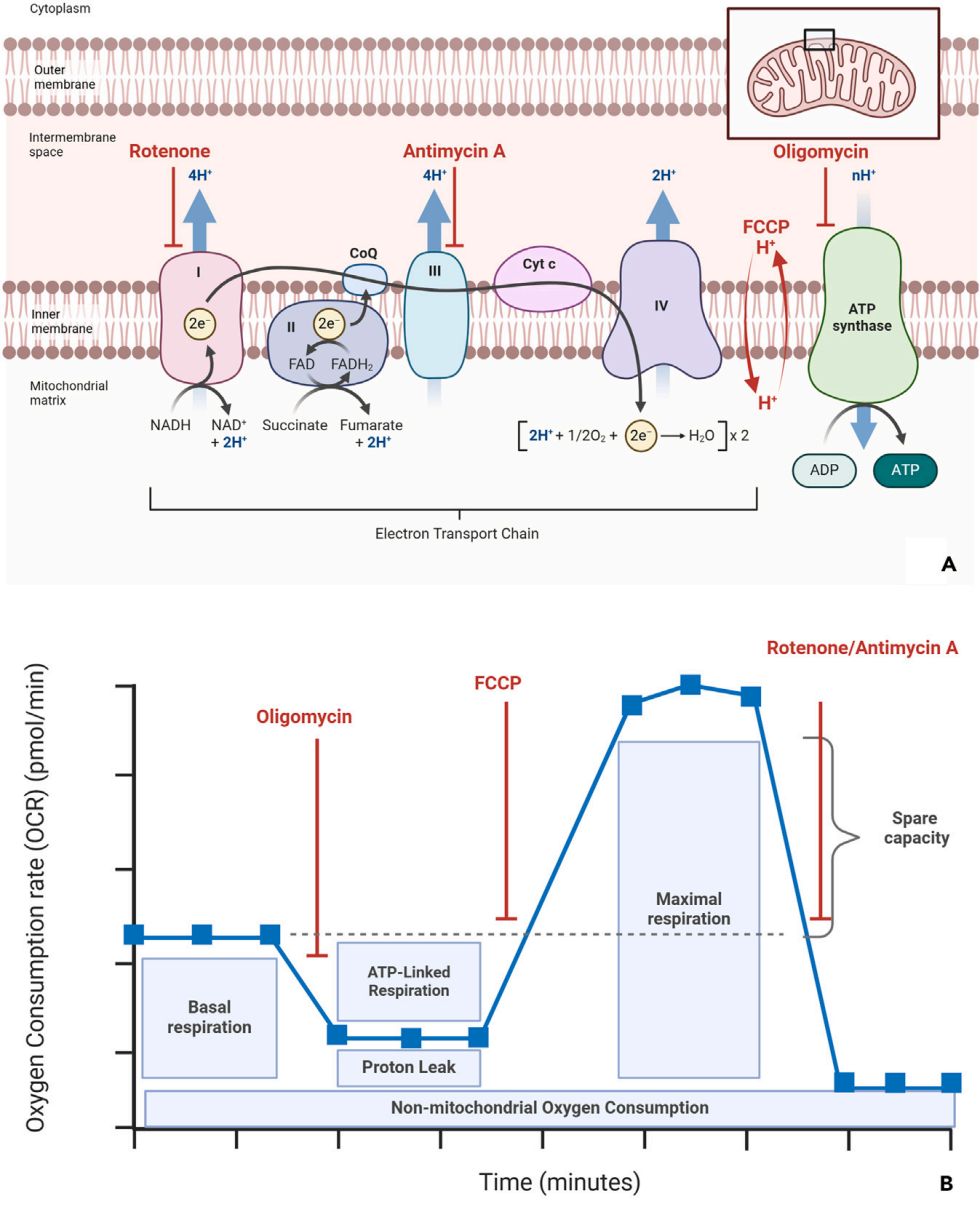
Illustrated scheme of mitochondrial function and Seahorse measurements Targets of inhibitors on the mitochondrial electron transport chain (ETC) (A). Key parameters of mitochondrial function measurable by Seahorse assays. (B). Oligomycin, an ATP synthase inhibitor, is injected first in the assay. It decreases electron flow through the ETC, reducing mitochondrial respiration or OCR. The second injection, FCCP, is an uncoupling agent that disrupts the mitochondrial membrane potential, allowing protons cross the membrane. This result in uninhibited electron flow through the ETC, with oxygen consumption by complex IV reaching its maximum. Spare respiratory capacity is defined as the difference between Maximal respiration and Basal respiration, representing the cell’s ability to meet increased energy demands or respond to stress. The third injection is a mixture of rotenone (a complex I inhibitor), and Antimycin A (a complex III inhibitor). This combination halts mitochondrial respiration, enabling the calculation of non-mitochondrial respiration, which reflects processes outside the mitochondria. Created in BioRender (https://BioRender.com/t05c186 and https://BioRender.com/s54t190).

For OCR data, calculate respiratory parameters such as basal respiration, maximal respiration, and spare capacity (Figures 6A and 6B). Non-mitochondrial oxygen consumption is derived from the lowest value after the final injection (e.g., 2-DG/rotenone/antimycin A). Basal respiration is calculated by subtracting the non-mitochondrial oxygen consumption value from the basal respiration value, and maximal respiration is similarly calculated by subtracting the non-mitochondrial oxygen consumption value from FCCP injection values. Spare capacity is determined by subtracting basal respiration from maximal respiration. Average values and standard deviations are calculated for replicates, with graphical representations of the data. Detailed calculations and examples are provided in File S1.

**Figure 7:**
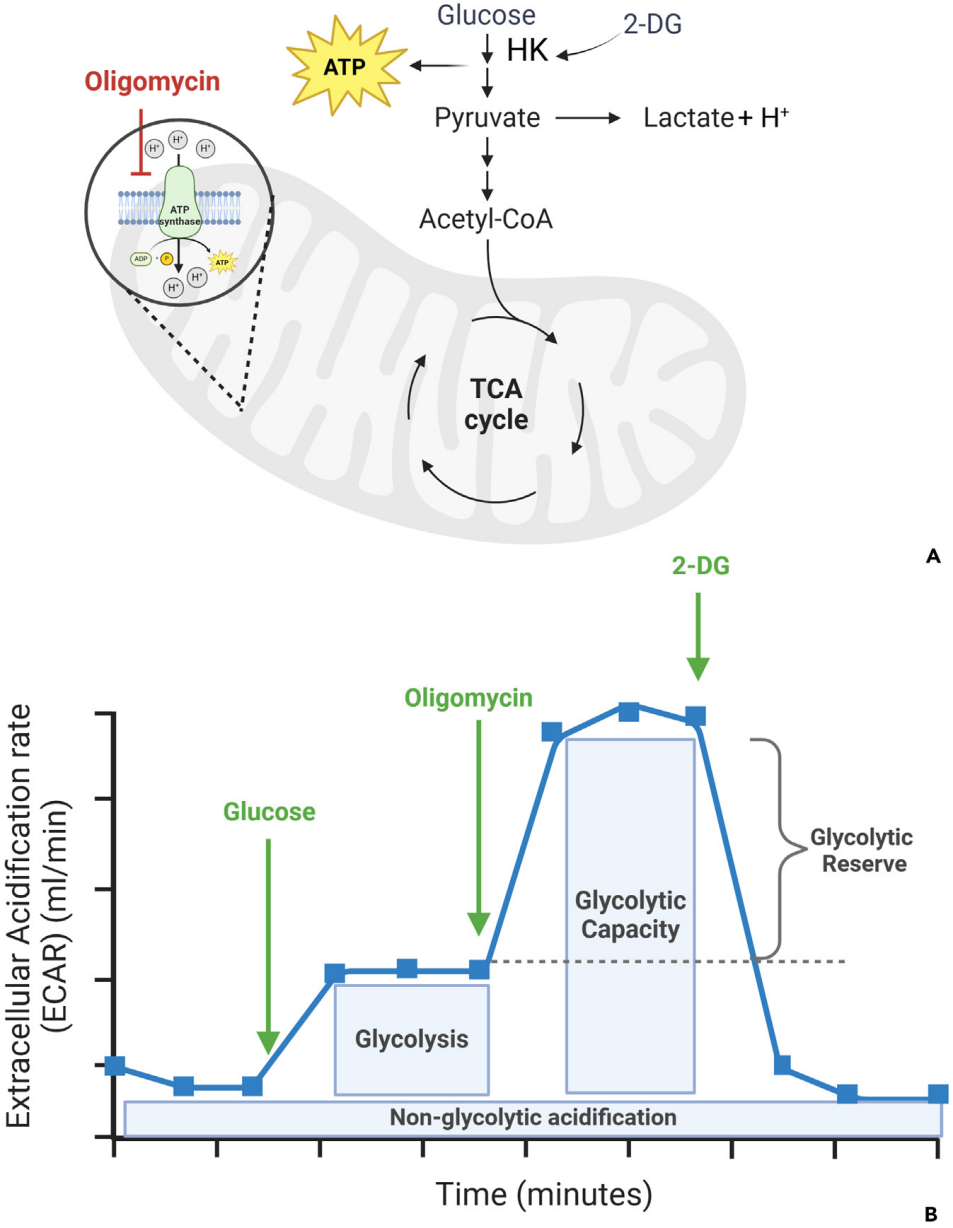
Illustrated scheme of Glycolysis and Seahorse measurement Sites of action of the added components in the glycolytic pathway (A). Key parameters of the glycolytic profile measurable by Seahorse assays (B). Glycolysis is driven by glucose addition, which enables the conversion of glucose to pyruvate and subsequently to lactate. This process produces protons that are extruded into the extracellular medium, leading to acidification and an increase in the ECAR. Glycolytic capacity is induced by blocking mitochondrial ATP production using a respiratory inhibitor, Oligomycin, which shifts energy production toward Glycolysis, further increasing ECAR. 2-DG, a glucose analog, inhibits Glycolysis by competitively binding to glucose hexokinase (HK) (first enzyme in the glycolytic pathway). The resulting decrease in ECAR confirms that the acidification observed is due to Glycolysis. The difference between Glycolytic capacity and Glycolysis rate defines the Glycolytic reserve. Non-glycolytic acidification is determined in the absence of glucose and is attributed to processes unrelated to Glycolysis. Created in BioRender (https://BioRender.com/m96t393 and https://BioRender.com/v33i467).

For ECAR data, analyze glycolytic parameters such as glycolysis, glycolytic capacity, and glycolytic reserve (Figures 7A and 7B). Non-glycolytic acidification is derived from the lowest value after the last injection (e.g., 2-DG/rotenone/antimycin A). Glycolysis is calculated by subtracting this value from the values obtained after glucose addition. Glycolytic capacity is calculated by subtracting non-glycolytic acidification from the oligomycin injection values, and glycolytic reserve is determined by subtracting glycolysis from glycolytic capacity. Averages and standard deviations are calculated, and the data is graphically represented. File S1 provides a detailed example of the calculations.

These analyses provide an overview of metabolic parameters for understanding the cellular metabolism. Data inclusion/exclusion should be based on the consistency of well readings and the exclusion of significant outliers.

### Intracellular ROS production

After completing kinetic measurement, save all data in an Excel file for analysis. First, calculate the average fluorescence intensity for wells without cells (blank control). Subtract this blank value from the fluorescence intensities of wells with treated cells to adjust for background fluorescence.

Then, calculate the average fluorescence intensity for each set of treatment group. Determine the percentage of ROS production for each value based on the fluorescence intensity and compute the average percentage for each treatment group. The final percentage values are used to create a graph showing ROS production differences across treatments. Detailed calculations and examples of this process can be found in File S2, which provides further guidance on how to perform these steps accurately.

### Result analysis of senescence

After collecting data, save results in Excel files for analysis. Since SA-β-Gal activity correlates with the protein content, start by calculating the ratio of fluorescence intensity (indicating SA-β-Gal activity) to the absorbance values (representing protein content from the BCA assay).

Once ratios are calculated, determine the average ratio for each treatment group. Next, calculate the percentage of SA-β-Gal production for each value and the average percentage for each group. Graph these final percentage values to illustrate differences in SA-β-Gal activity across various treatments. For detailed examples and calculations, refer to File S3, which provides a clear outline of the steps to ensure accurate and reproducible results.

## Limitations

Working with primary hBMSCs presents several limitations that researchers have to consider. Ethical approval is essential when collecting human BM aspirates. Additionally, biological variability due to factors such as age, sex, genetic background, and health status of the donors can affect reproducibility and complicate data interpretation. Results from one donor may not replicate with another, posing a challenge in developing standardized protocols. The quantity and quality of the BM aspirates can also vary, affecting the yield and viability of isolated hBMSCs. Low sample quality can result in reduced cell numbers or impair cell function. Limited cell count from clinical samples may restrict the number of assays that can be performed, often requiring prioritization of specific assays. Advanced techniques for assessing cellular metabolism, such as bioenergetics profiling, ROS production, and senescence assays, often require specialized equipment, technical expertise, and meticulous optimization. Even minor variations in protocol performance can significantly impact the results.

**Figure 8:**
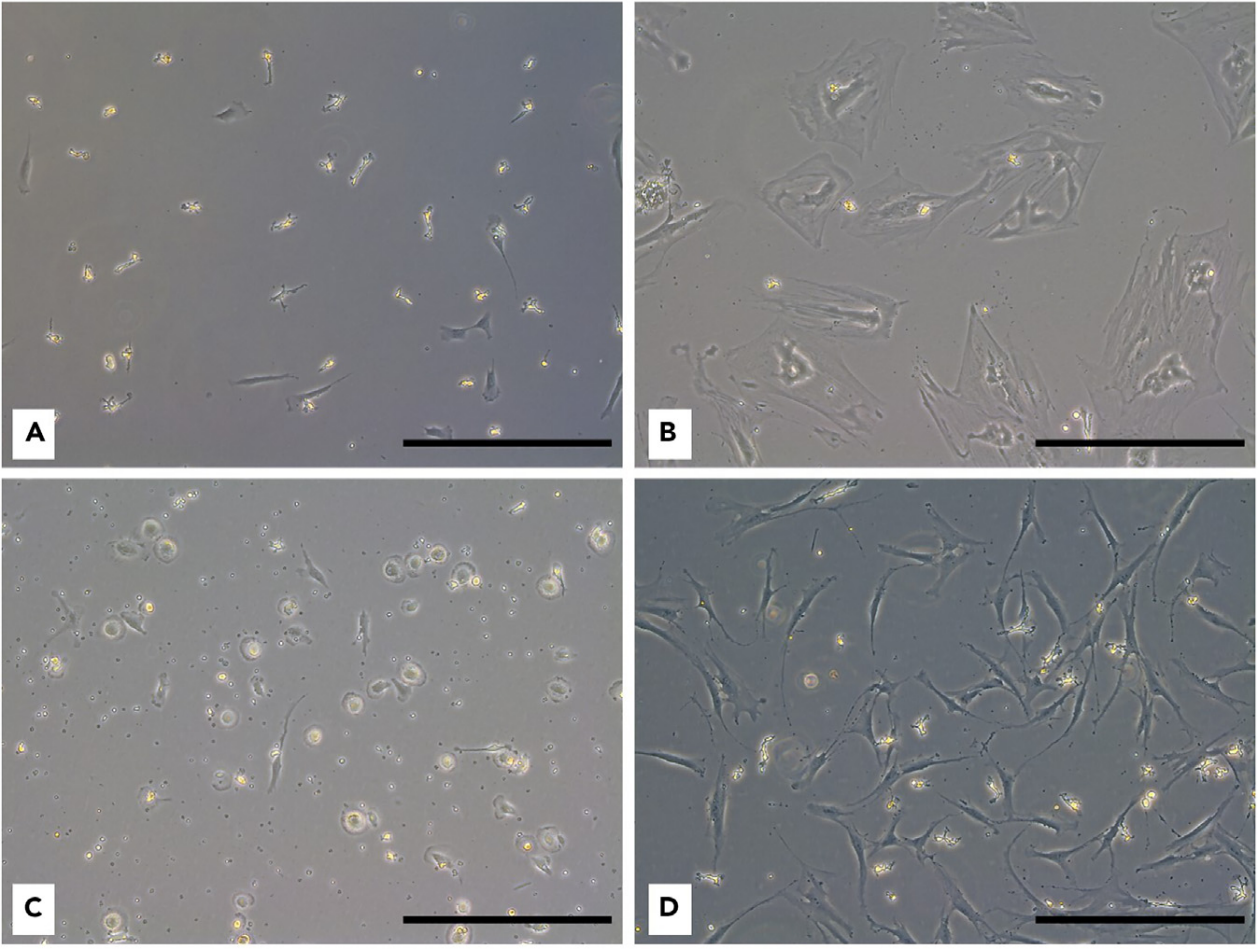
Different morphology of hBMSCs (A–D) Scale bar: 200 μm, magnification: 10×.

**Figure 9:**
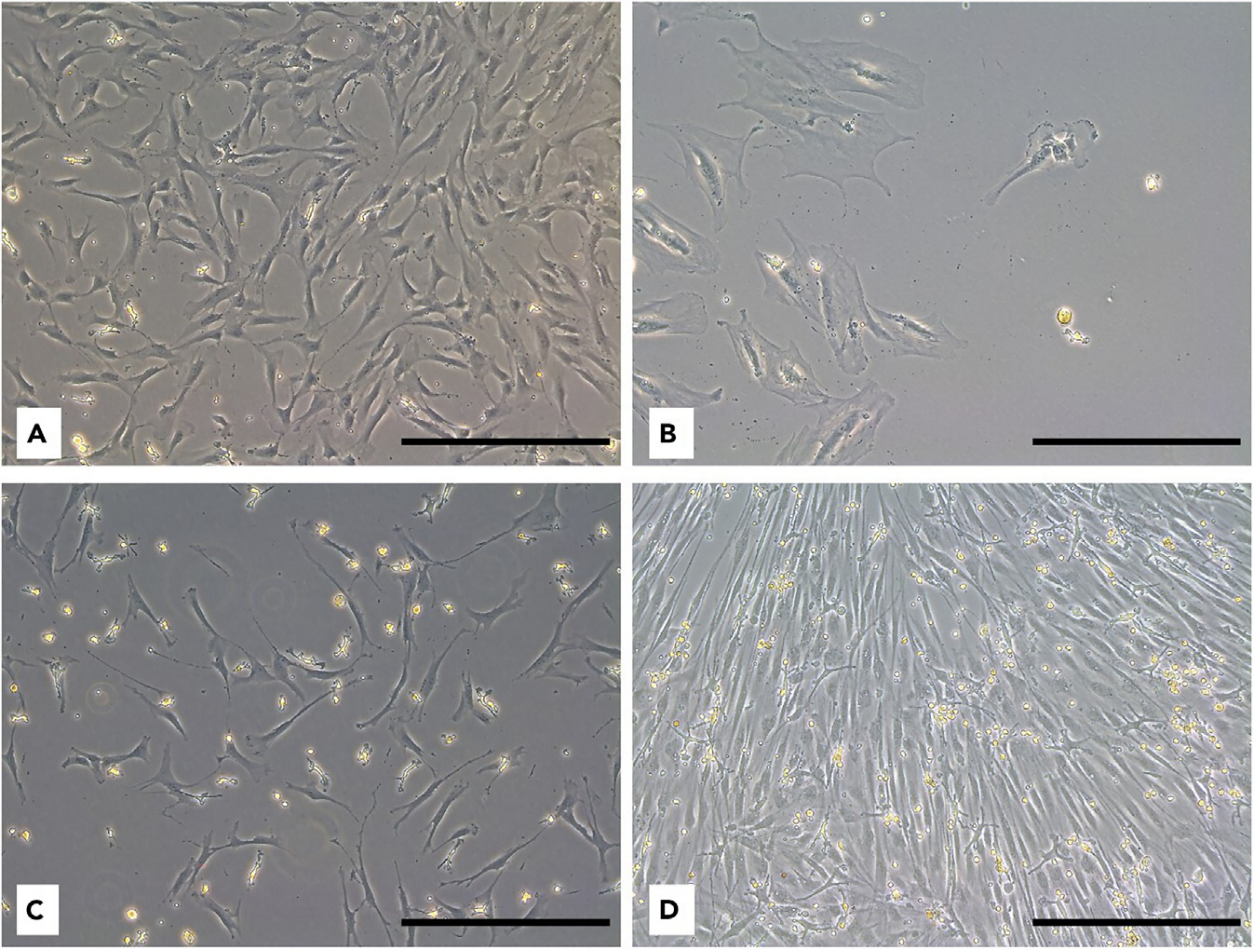
Different confluence of hBMSCs Optimal confluence is 80–90% (A); Confluence is less than 50% (B); Confluence is approx. 50% (C); Cells are over confluent (D). Scale bar: 200 μm, magnification: 10×.

## Troubleshooting

### Problem 1

hBMSCs do not grow uniformly.

### Potential solution 1

• Cell size: Primary hBMSCs are heterogeneous population of cells. Therefore, it is important to consider their varying sizes before seeding for experiments (Figures 8A–8D). We recommend to try different seeding density to optimize the experiment outcome.
• Cell confluence: Maintain cell density at approx. 80–90% to ensure healthy status of cell cultures (Figure 9A). Incorrect seeding density or uneven distribution can negatively impact cell metabolism and lead to inconsistent results (Figures 9B, 9C, and 9D).

### Problem 2

hBMSCs exhibit low viability after freezing.

### Potential solution 2

Long-term storage in liquid nitrogen halts metabolic processes and preserves cell viability for years. Upon thawing, cells may exhibit altered metabolic activity as they recover from the stress of freezing. However, providing optimal recovery conditions, such as a well-prepared growth medium and avoiding immediate stress (e.g., passaging), helps cells to regain their metabolic balance. Perform as many measurements as possible to avoid freezing cells. When freezing is necessary, follow precise protocols to ensure the cells are processed the same way. Freezing can impact cell viability and metabolism, depending on the freezing protocol, storage conditions, and cell type. Use cryoprotectant, DMSO, which helps to prevent ice crystal formation and minimizes cellular dehydration. Avoid repeated freeze-thaw cycles by using each aliquot only once.

### Problem 3

Inconsistent seahorse OCR or ECAR measurements.

### Potential solution 3

• Cell seeding: Ensure even cell distribution across all wells to avoid variability in OCR/ECAR measurements. Uneven cell distribution can cause inconsistent results. Test and optimize the seeding density for each cell type before starting experiments. Avoid over-confluent cells, as this can impair proper respiration. Before the first measurement, test different densities to adjust the experimental setup.
• Assay optimization: Verify that assay reagents are freshly prepared and the protocol is optimized for your cell type. Perform control experiments to ensure proper assay conditions.

### Problem 4

Low signals close to the background in seahorse OCR/ECAR data.

### Potential solution 4

Insufficient cell numbers can lead to low signal detection. Increase the number of cells seeded per well, ensuring they are not over-confluent, which could alter the metabolic state.

### Problem 5

Shift in baseline Seahorse OCR/ECAR measurements.

### Potential solution 5

Ensure the incubator and analyzer are pre-warmed and equilibrated to the correct temperature and CO_2_ levels before starting the assay. Any fluctuations can cause shift in the baseline measurements.

### Problem 6

Low or no response to mitochondrial stress test compounds in Seahorse measurement.

### Potential solution 6

• Compound degradation: Check the expiration date and storage conditions of compounds like oligomycin, FCCP, and rotenone. Degraded compounds may not elicit the expected response. Use fresh working solutions prepared as recommended.
• Insufficient concentrations: Confirm that the concentrations used are high enough to stimulate cells. Perform a control experiment to optimize reagent concentration and protocol setup.

### Problem 7

Poor response to inhibition of ATP production in Seahorse measurement.

### Potential solution 7

Optimize the oligomycin concentration for your cell type. Too low concentration may not effectively inhibit ATP synthase. Perform a dose-response curve to determine the optimal concentration.

### Problem 8

Respiration observed in background wells before data normalization in Seahorse measurement.

### Potential solution 8

The detection of a signal in background wells may indicate the presence of cells or contamination. Ensure background wells contain no cells. You can prevent this by properly labeling the plate lid before pipetting cells into the cell plate.

### Problem 9

Unexpected pH changes (e.g., pH < 7.4 or unstable pH during the Seahorse measurement).

### Potential solution 9

• pH temperature sensitivity: Protect Seahorse plates from cooling. Minimize the time when the plates are outside the incubator to prevent the media and plastic from cooling down.
• Decreasing pH during the measurement: High cell numbers within the wells may cause pH to drop. Optimize your seeding density as outlined in Potential solution 1.

### Problem 10

High background fluorescence in ROS assays.

### Potential solution 10

High background fluorescence may result from excessive dye concentration or prolonged incubation. To minimize non-specific binding and auto-fluorescence, optimize the concentration of the ROS-sensitive dye (e.g., DCFDA) and reduce the incubation time.

### Problem 11

Rapid quenching of fluorescence signal during ROS production measurement.

### Potential solution 11

ROS signals can diminish quickly. To address this problem, prepare the plate reader or flow cytometer in advance and begin measurement immediately after adding the dye.

### Problem 12

Overestimation of ROS levels due to autofluorescence.

### Potential solution 12

To account for autofluorescence, include unstained controls and subtract their signal from the ROS-specific signal during data analysis.

### Problem 13

Fluorescence signal is very low or inconsistent between measurements.

### Potential solution 13

If the entire kit is not used in a single measurement, it is highly recommended to aliquot the DCFDA and TBHP solutions properly. Before each measurement, thaw only required amount of these solutions. Avoid repeated freeze-thaw cycles, as this can negatively impact the fluorescence signal and lead to inconsistent results.

### Problem 14

Overestimation of signal leading to false-positive results.

### Potential solution 14

• High variability: Cellular senescence is highly complex phenotype influenced by multiple factors. The choice of biomarker derived from the hallmarks of cellular senescence should be carefully tailored to the specific context and cell type.
• High fluorescence: Overestimation can result from autofluorescence of the cells, excessive dye concentration, or prolonged incubation. No individual biomarker can identify cellular senescence on its own, as such markers are not uniquely specific to the senescent state. This limitation often results in low detection rates (10–15%) for senescence biomarkers. To improve detection accuracy, use a multi-biomarker approach or simultaneously assess multiple hallmarks of senescence.

### Problem 15

Fluorescence signal is very low or inconsistent between measurements.

### Potential solution 15

Properly aliquot the 20× SA-ß-Gal Substrate to avoid repeated freeze-thaw cycles, as this can negatively impact fluorescence signal and lead to inconsistent results. Additionally, ensure that all kit components are within their expiration date before use.

## Resource availability

### Lead contact

Further information and requests for resources and reagents should be directed to and will be fulfilled by the lead contact, Michaela Tencerova (Michaela.tencerova@fgu.cas.cz).

### Technical contact

Further information and requests for resources and reagents should be directed to and will be fulfilled by the technical contact, Michaela Tencerova (Michaela.tencerova@fgu.cas.cz).

### Materials availability

This study did not generate new unique reagents.

### Data and code availability

This study did not generate any unique datasets or code. The example of possible outcome of the measurements is provided in the Supplementary Material.

## Acknowledgments

We thank Tomas Mracek, Alena Pecinova, and Olga Zimmermanova for their excellent assistance with the establishment of the Seahorse protocol and Cytation measurement at IPHYS, Prague. We are grateful for the enormous work of our present and former colleagues from KMEB, University of Southern Denmark, Odense, and IPHYS, Prague, who contributed to the establishment and optimalization of the protocols presented here. We are extremely grateful to all clinicians, surgeons, nurses, and patients who were and are actively engaged in the collection of the clinical samples, and this research would not have been possible without their dedication. The graphical abstract was created with BioRender.com (https://BioRender.com/t61y299). This work was supported by the Czech Science Foundation (GACR 20-03586S and GACR 22-12243S) (M.T.), the EFSD/Novo Nordisk Foundation Future Leaders Award (NNF20SA0066174) (M.T.), the National Institute for Research of Metabolic and Cardiovascular Diseases (program EXCELES, ID project no. LX22NPO5104) funded by the European Union – Next Generation EU, the Ministry of Health of the Czech Republic (NU23-01-00125) (M.T.), and the Intra-Visegrad Scholarship (52310168) (M.D.).

## Author contributions

M.T. and M.D. conceived the project. M.D., M.F., A.B., D.A., and M.T. designed the experiments, analyzed data, and wrote the manuscript. All authors revised and approved the manuscript.

## Declaration of interests

The authors declare no competing interests.

## Supplemental information

File S1. Example of calculation for Seahorse measurement, related to Quantification and statistical analysis section (A) OCR evaluation. (B) ECAR evaluation.

File S2. Example of calculation for ROS production measurement, related to Quantification and statistical analysis section

File S3. Example of calculation for Senescence measurement, related to Quantification and statistical analysis section
